# Effect of human umbilical cord blood mesenchymal stem cell transplantation on neuronal metabolites in ischemic rabbits

**DOI:** 10.1186/1471-2202-15-41

**Published:** 2014-03-17

**Authors:** Ye-Ming Guan, Yao Zhu, Xue-Chun Liu, Hai-Li Huang, Zhi-Wei Wang, Bo Liu, You-Zi Zhu, Qing-Song Wang

**Affiliations:** 1 Department of Neurology, The 105th Hospital of PLA, Clinic College, Anhui Medical University, Hefei 230031, China; 2 Center for Cell Therapy, The 105th Hospital of PLA, Clinic College, Anhui Medical University, Hefei 230031, China; 3 Department of Radiology, The 105th Hospital of PLA, Clinic College, Anhui Medical University, Hefei 230031, China

**Keywords:** Human umbilical cord blood mesenchymal stem cells, Cerebral ischemia, Proton magnetic resonance spectroscopy, Middle cerebral artery occlusion

## Abstract

**Background:**

Because there is little research on the effects of transplanted stem cells on neuronal metabolites in infarct areas, we transplanted human umbilical cord blood mesenchymal stem cells (hUCB-MSCs) into cerebral ischemic rabbits and examined the neuronal metabolites.

**Results:**

Rabbits (n = 40) were equally divided into sham, middle cerebral artery occlusion (MCAO), hUCB-MSC, and saline groups. The rabbit ischemic model was established by MCAO. The effects of hUCB-MSC transplantation were assessed by proton magnetic resonance spectroscopy (^1^H-MRS), neurological severity scores (NSSs), infarct area volume, neuronal density, and optical density (OD) of microtubule-associated protein 2 (MAP2)-positive cells. We also evaluated complete blood cell counts(CBCs) and serum biochemical parameters. NSSs in the hUCB-MSC group at 7 and 14 days after reperfusion were lower than in MCAO and saline groups (*p* < 0.05). Compared with MCAO and saline groups at 2 weeks after MCAO, the infarction volume in the hUCB-MSC group had decreased remarkably (*p* < 0.05). Significant neuronal metabolic changes occurred in the infarct area at 24 h and 2 weeks after MCAO. ^1^H-MRS revealed an elevation in the lactate (Lac)/creatine including phosphocreatine (Cr) ratio and a decrease in the N-acetylaspartate (NAA)/Cr and choline-containing phospholipids (Cho)/Cr ratios at 24 h after MCAO in the MCAO group (*p* < 0.01). Compared with saline and MCAO groups at 24 h and 2 weeks after MCAO, NAA/Cr and Cho/Cr ratios had increased significantly, whereas the Lac/Cr ratio had decreased significantly in the hUCB-MSC group (*p* < 0.01). Neuronal density and OD of MAP2-positive cells in the MCAO group were significantly lower than those in the sham group, whereas the neuronal density and OD of MAP2-positive cells in the hUCB-MSC group were higher than those in MCAO and saline groups (*p* < 0.05). CBCs and biochemical parameters were unchanged in the MCAO group at 24 h and 2 weeks after hUCB-MSC transplantation.

**Conclusions:**

Transplanted hUCB-MSCs might ameliorate ischemic damage by influencing neuronal metabolites in the infarct area, providing additional evidence for neuroprotection by stem cells. No significant changes were observed in CBCs or serum biochemical parameters, suggesting that intravenous infusion of hUCB-MSCs is safe for rabbits in the short-term.

## Background

Cerebral vascular diseases are a major cause of mortality and disability worldwide. In China, stroke is a leading cause of death and the most common cause of adult disability. At least 1,500,000 patients are affected by cerebrovascular diseases annually, and approximately 30% of these patients become severely and permanently disabled. Many other survivors are left with permanent functional impairments [1]. Some interventions during the acute phase of stroke such as thrombolytic and neuroprotective agents have been recognized to improve patient outcomes including survival and residual disability. However, once cell damage from stroke has been established, little can be done to restore pre-stroke conditions. In this regard, hundreds of studies have described the therapeutic potential of either endogenous or transplanted stem cells in experimental models of stroke [2]. Therefore, elucidation of potential mechanisms of action is vital to further develop stem cell therapies for translation to the bedside. Various proposed mechanisms have been investigated in preclinical stroke models, including formation of new neuronal circuitry, reductions of apoptosis and inflammation, and promotion of angiogenesis, neurogenesis, and other endogenous repair processes [3-6].

Proton magnetic resonance spectroscopy (^1^H-MRS) is a non-invasive method to detect various neuronal metabolites in real time, including N-acetylaspartate (NAA), creatine including phosphocreatine (Cr), choline-containing phospholipids (Cho), and lactate (Lac) [7]. These advances have led to the emergence of novel approaches to pathophysiological studies of cerebral ischemia [8]. However, little has been reported about how transplanted stem cells might change neuronal metabolites in the infarct area. Thus, we hypothesized that transplanted stem cells may ameliorate ischemic damage by influencing neuronal metabolites in the infarct area. To test this hypothesis, we used a rabbit ischemic model of middle cerebral artery occlusion (MCAO) for transplantation of human umbilical cord blood mesenchymal stem cells (hUCB-MSCs). We then assessed neurological severity scores (NSSs), infarct area volumes, neuronal density, and optical density (OD) of microtubule-associated protein-2 (MAP2)-positive cells. NAA, Cr, Cho, and Lac in the infarct region were measured by ^1^H- MRS. We also obtained complete blood cell counts (CBCs) and measured serum biochemical parameters.

## Results

### Phenotype of hUCB-MSCs

**Figure 1:**
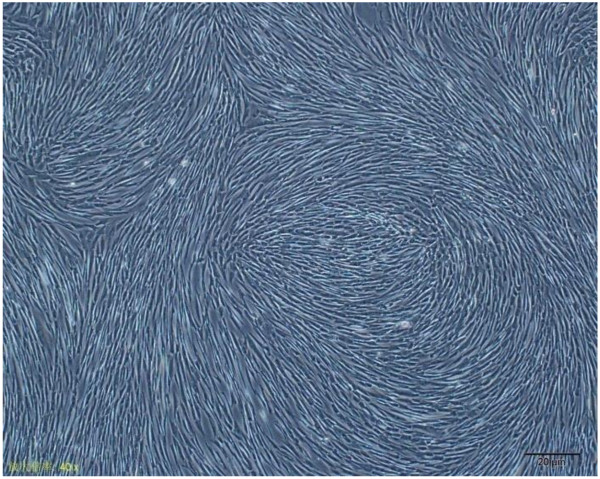
**Morphological features of MSCs isolated from hUCB.** Passage 5 hUCB-MSCs subcultured for 7 days. The cells exhibited a fusiform or fibroblast-like shape, and were arranged as a whirlpool. Magnification, ×40.

The cells picked from clones could be passaged successfully and the cells grew as a fusiform or fibroblast-like shape, whereas the subclones developed like a whirlpool (Figure 1).

**Figure 2:**
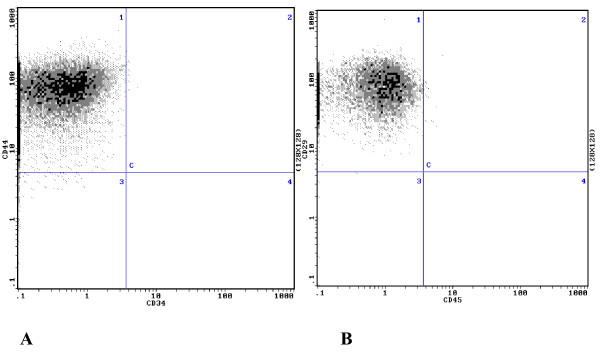
**Detection of surface marker expression in hUCB-MSCs by flow cytometry. A**, 98.9 ± 1.41% CD44^+^ cells and 1.2 ± 0.34%CD34^+^ cells. **B**, 98.7 ± 1.31% CD29^+^ cells and 1.3 ± 0.31% CD45^+^ cells.

Isolated hUCB-MSCs were characterized by flow cytometric analysis of specific surface antigens. hUCB-MSCs expressed surface antigens CD29 and CD44, but not CD34 and CD45, which was consistent with the surface antigens of bone marrow-derived MSCs (Figure 2).

### hUCB-MSC transplantation affects NSSs and the infarct volume in MCAO rabbits

**Figure 3:**
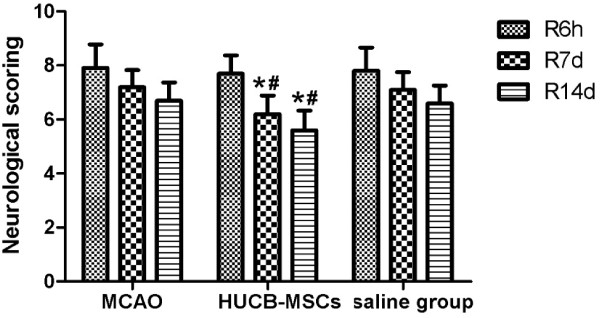
**Effect of hUCB-MSC transplantation on NSSs in MCAO rabbits (n = 10, mean ± SE).** *p < 0.05 versus the MACO group; ^#^*p* < 0.05 versus the saline group.

To evaluate the effect of hUCB-MSCs on neurological deficits after MCAO, we measured NSSs. The NSSs in the hUCB-MSC group at 7 and 14 days after reperfusion were less than those in MACO and saline groups (*p* < 0.05) (Figure 3).

**Figure 4:**
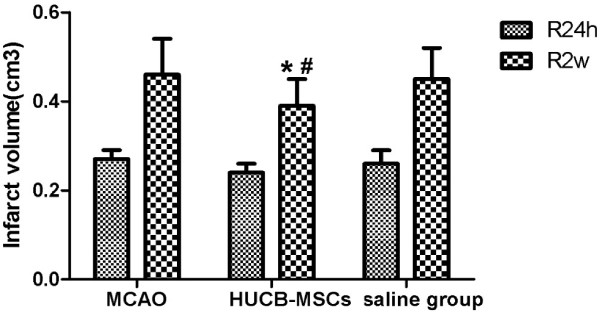
**Effect of hUCB-MSC transplantation on the infarct volume in MCAO rabbits (n = 10, mean ± SE).** *p < 0.05 versus the MACO group; ^#^*p* < 0.05 versus the saline group.

Compared with MCAO and saline groups at 2 weeks after MCAO, infarct volumes in the hUCB-MSC group decreased remarkably (*p <* 0.05) (Figure 4).

### hUCB-MSC transplantation affects neuronal metabolites in MCAO rabbits

**Figure 5:**
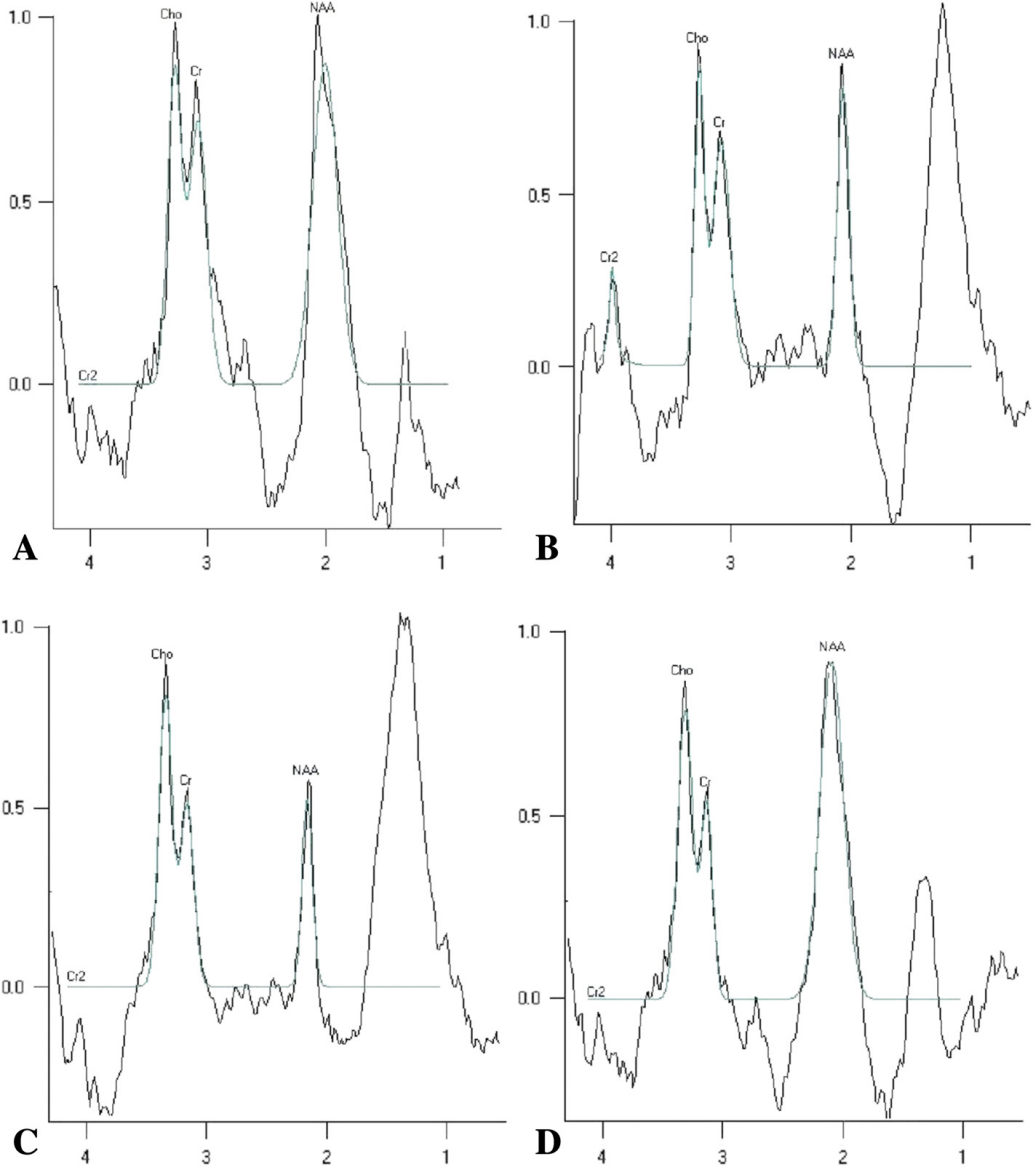
^**1**^**H-MRS spectra of the various groups. A) **^1^H-MRS spectra in the sham group had upright steep peaks of NAA and Cho. A Lac peak was not detected. **B) **^1^H-MRS spectra in the MCAO group revealed a reduction in the NAA peak and distinct upright, steep peaks of Lac were detected at 24 h after MCAO. **C) **^1^H-MRS spectra in the saline group revealed a reduction in the NAA peak. Similar to the saline group, upright steep peaks of Lac were detected at 24 h after MCAO. **D) **^1^H-MRS spectra in the hUCB-MSC group at 24 h after MCAO. Compared with MCAO and saline groups, the NAA peak was higher and the Lac peak was lower.

**Figure 6:**
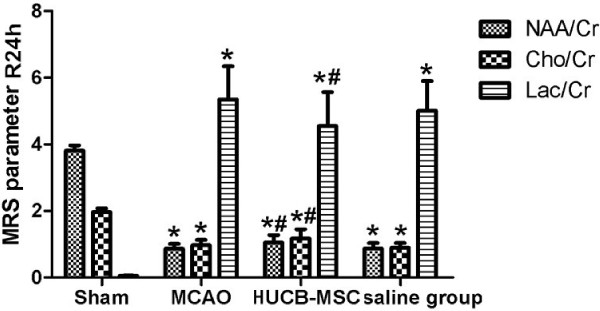
**Effect of hUCB-MSC transplantation on **^**1**^**H-MRS parameters at 24 h after MCAO (n = 10, mean ± SE).** *p < 0.01 versus the sham group; ^#^*p* < 0.01 versus MCAO and saline groups.

**Figure 7:**
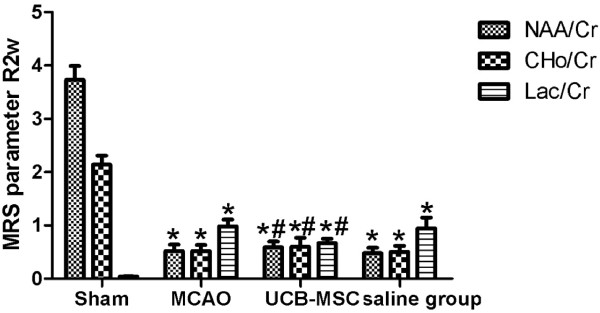
**Effect of hUCB-MSC transplantation on **^**1**^**H-MRS at 2 weeks after MCAO (n = 10, mean ± SE).** *p < 0.01 versus the sham group; ^#^*p* < 0.01 versus MCAO and saline groups.

To observe the effects of hUCB-MSC transplantation on neuronal metabolites in the infarct area, ^1^H-MRS was used to detect the ratios of NAA/Cr, Cho/Cr ,and Lac/Cr at 24 h and 2 weeks after MCAO. Significant neuronal metabolic changes in the infarct area were observed at 24 h and 2 weeks after MCAO (Figure 5). ^1^H-MRS spectra showed significant elevation in the Lac/Cr ratio and a marked decrease in NAA/Cr and Cho/Cr ratios at 24 h after MCAO in the MCAO group (*p* < 0.01 versus the sham group). Compared with saline and MCAO groups at 24 h after MCAO, NAA/Cr and Cho/Cr ratios were significantly increased and the Lac/Cr ratio was significantly decreased in the hUCB-MSC group (*p* < 0.01) (Figure 6). Interestingly, at 2 weeks after MCAO, NAA/Cr and Cho/Cr ratios had increased substantially in the hUCB-MSC group, whereas the Lac/Cr ratio has decreased remarkably compared with those in saline and MCAO groups (*p* < 0.01) (Figure 7).

### Distribution and migration of hUCB-MSCs in the injured brain

**Figure 8:**
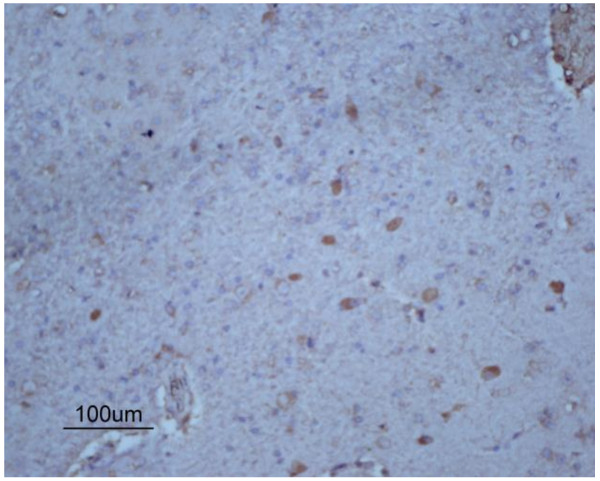
**Representative photomicrographs (×100) of hUCB-MSCs in the injured brain.** BrdU-positive cells were within the ischemic area.

To investigate whether transplanted hUCB-MSCs could migrate to the infarcted brain area, hUCB-MSCs labeled with 5-bromodeoxyuridine (BrdU) were injected via the right femoral vein of rabbits in the hUCB-MSC group. BrdU-positive cells migrated selectively to the infarction area, which were small with round or oval nuclei containing intensely stained brown chromatin (Figure 8).

### hUCB-MSC transplantation affects neuronal density and OD of MAP2-positive cells in the infarct area

**Figure 9:**
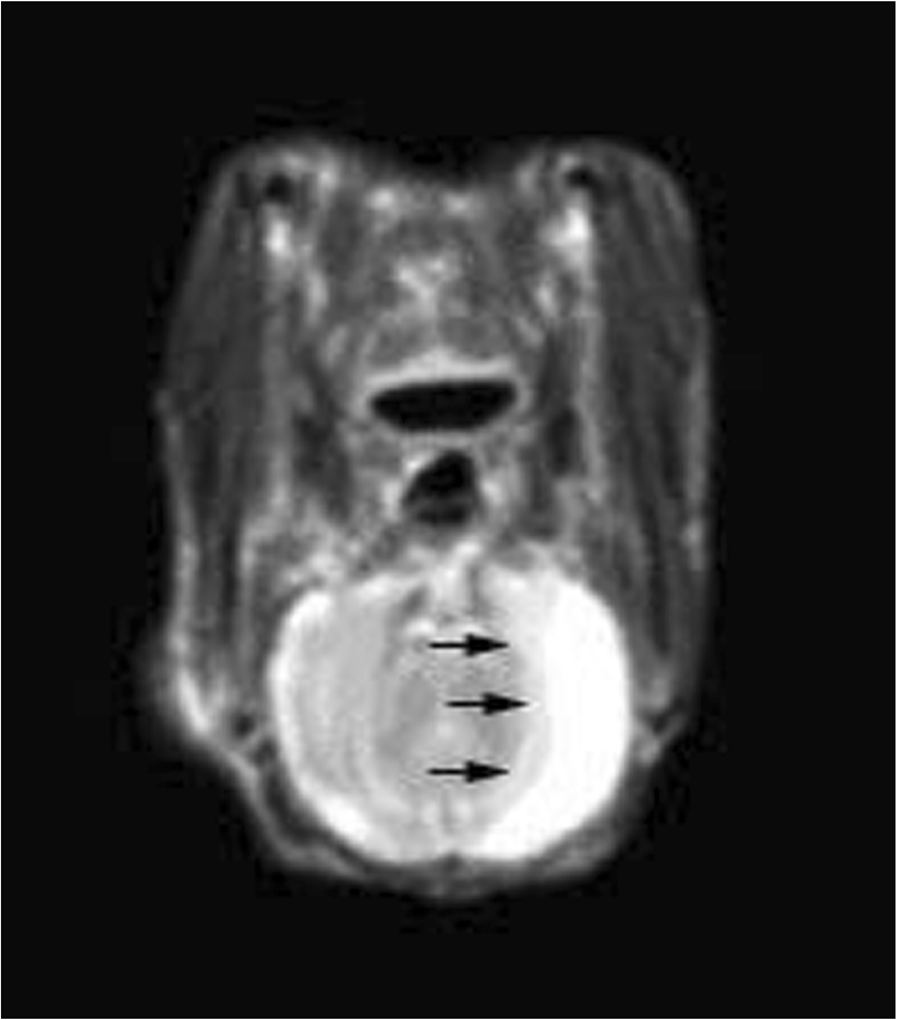
**Representative head MRI of MCAO model rabbits.** At 2 days after MCAO, T2WI revealed a hyperintensity in the area of the middle cerebral artery. Black arrows indicate the ischemic area.

**Figure 10:**
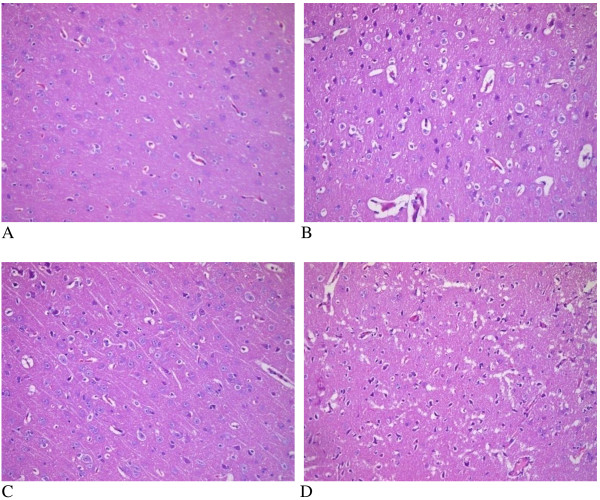
**Representative photomicrographs (×200) of the infarct area. A**, sham group; **B**, MCAO group; **C**, hUCB-MSC group; **D**, saline group.

**Figure 11:**
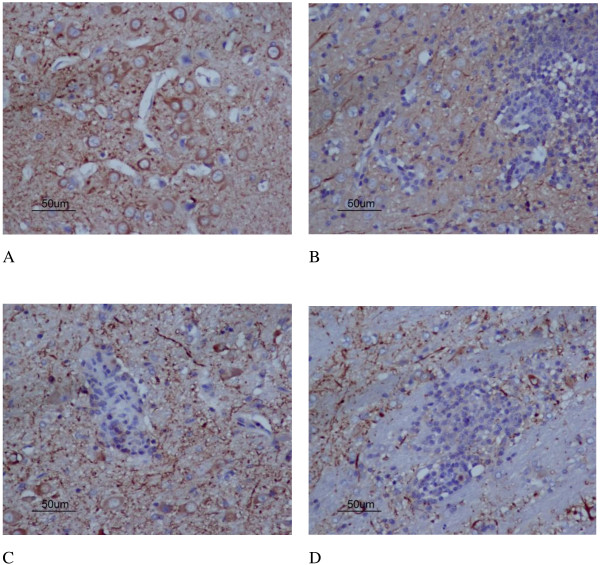
**Representative photomicrographs (×200) of MAP2 immunohistochemistry. A**, sham group; **B**, MCAO group; **C**, hUCB-MSC group; **D**, saline group.

**Figure 12:**
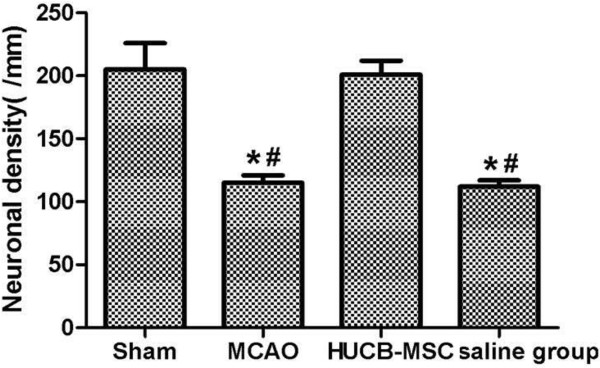
**Neuronal density (per mm**^**2**^**) among groups (n = 10, mean ± SE).** *p < 0.05 versus the sham group; ^#^*p* < 0.05 versus MCAO and saline groups.

**Figure 13:**
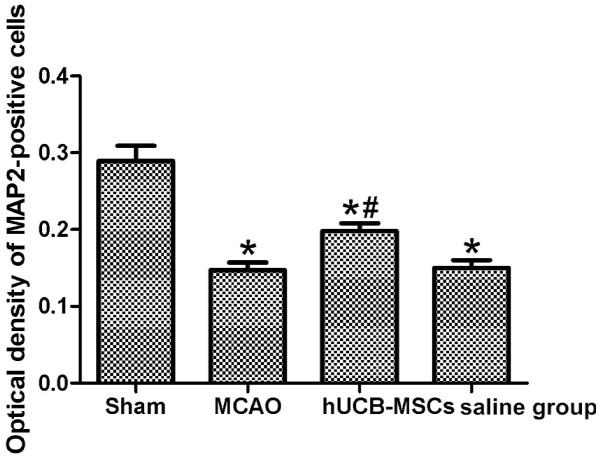
**Optical density of MAP2-positive cells among groups (n = 10, mean ± SE).** *p < 0.05 versus sham controls; ^#^*p* < 0.05 versus MCAO and saline groups.

MCAO can cause a complete cerebral infarction as shown by magnetic resonance imaging (MRI) (Figure 9). Cell morphology in the sham group was normal with only a few necrotic cells. More necrotic cells were observed in MCAO and saline groups (Figure 10). MAP2 immunostaining products were localized within the cytodendrites of brain neurons in the sham group. MAP2 immunolabeling was markedly diminished in the infarct zone, and there was an abrupt transition between the infarct and adjacent normal tissue with occasional MAP2-positive cells in the transition zone (Figure 11). To investigate whether transplanted hUCB-MSCs could protect neurons, we examined neuronal density and OD of MAP2-positive cells in the infarct area. Neuronal density and OD of MAP2-positive cells in the MCAO group were significantly lower than those in the sham group (*p* < 0.05). However, neuronal density and OD of MAP2-positive cells in the hUCB-MSC group was higher than those in MCAO and saline groups (*p* < 0.05) (Figures 12 and 13). These data suggest that extensive neuronal damage occurs after MCAO and hUCB-MSC transplantation can prevent ischemic damage.

### Effects of hUCB-MSC transplantation on CBCs and serum biochemical parameters in MCAO rabbits

To investigate the safety of hUCB-MSC transplantation in rabbits, CBCs and serum biochemical parameters were measured at 24 h and 2 weeks after MCAO. We found no influence on CBCs, liver function, renal function, serum glucose, or serum lipids in MCAO rabbits at 24 h and 2 weeks after transplantation (Tables 1, 2, 3, 4).

**Table 1:** CBCs of each group at 24 h after MCAO (n = 10, mean ± SE)

	**WBCs (10**^**9**^**/L)**	**RBCs (10**^**12**^**/L)**	**HB (g/L)**	**PLT (10**^**9**^**/L)**
Sham	7.56 ± 1.40	5.05 ± 0.55	120.50 ± 17.04	241.90 ± 56.58
MCAO	7.74 ± 1.16	5.36 ± 0.73	115.00 ± 14.94	258.00 ± 68.81
hUCB-MSC	7.50 ± 1.30	5.22 ± 0.70	112.10 ± 13.07	281.30 ± 88.51
Saline	7.50 ± 1.92	5.33 ± 0.62	119.60 ± 20.80	240.50 ± 63.31

Note: white blood cells (WBCs); red blood cells (RBCs); hemoglobin (HB); platelet count (PLT).

**Table 2:** Serum biochemistry of each group at 24 h after MCAO (n = 10, mean ± SE)

	**Sham**	**MCAO**	**hUCB-MSC**	**Saline**
ALT (U/L)	88.30 ± 24.74	85.20 ± 22.95	95.40 ± 22.20	88.80 ± 17.93
AST (U/L)	90.50 ± 6.85	95.20 ± 18.82	95.40 ± 18.39	90.00 ± 15.89
Urea (mmol/L)	5.93 ± 1.03	6.06 ± 0.95	5.68 ± 1.26	5.65 ± 1.03
Cr (μmol/L)	90.20 ± 24.89	86.50 ± 12.87	92.10 ± 14.43	94.70 ± 11.69
TG (mmol/L)	0.84 ± 0.24	1.11 ± 0.81	1.18 ± 0.83	1.26 ± 1.12
Chol (mmol/L)	1.89 ± 0.75	1.57 ± 0.83	1.50 ± 0.74	1.74 ± 0.57
Glu (mmol/L)	8.07 ± 0.91	7.98 ± 0.85	8.44 ± 1.35	8.41 ± 1.47

Note: aspartatenine aminotransferase (ALT); aspartate aminotransferase (AST).

**Table 3:** CBCs of each group at 2 weeks after MCAO (n = 10, mean ± SE)

	**WBCs (10**^**9**^**/L)**	**RBCs (10**^**12**^**/L)**	**HB (g/L)**	**PLT (10**^**9**^**/L)**
Sham	7.32 ± 1.28	5.21 ± 0.46	122.52 ± 14.07	245.60 ± 53.21
MCAO	7.57 ± 1.41	5.62 ± 0.23	114.40 ± 13.26	255.00 ± 70.07
hUCB-MSC	7.28 ± 1.08	5.34 ± 0.41	112.06 ± 13.64	277.30 ± 85.65
Saline	7.33 ± 1.15	5.77 ± 0.52	117.63 ± 18.83	270.50 ± 82.77

Note: white blood cells (WBCs); red blood cells (RBCs); hemoglobin (HB); platelet count (PLT).

**Table 4:** Serum biochemistry of each group at 2 weeks after MCAO (n = 10, mean ± SE)

	**Sham**	**MACO**	**hUCB-MSC**	**Saline**
ALT (U/L)	78.40 ± 21.12	82.50 ± 24.05	88.80 ± 23.36	90.50 ± 19.34
AST(U/L)	89.60 ± 16.75	91.70 ± 18.52	92.40 ± 17.45	92.20 ± 15.79
Urea (mmol/L)	5.71 ± 1.05	5.56 ± 0.87	5.78 ± 1.03	5.47 ± 0.76
Cr (μmol/L)	87.20 ± 15.43	89.86 ± 15.34	90.70 ± 14.23	92.40 ± 13.64
TG (mmol/L)	0.75 ± 0.17	1.25 ± 0.61	1.27 ± 0.47	1.04 ± 0.26
Chol (mmol/L)	1.76 ± 0.86	1.79 ± 0.45	1.67 ± 0.38	1.58 ± 0.62
Glu (mmol/L)	7.47. ± 0.91	8.03 ± 0.25	8.23 ± 1.27	7.61 ± 0.83

Note: aspartatenine aminotransferase (ALT); aspartate aminotransferase (AST).

## Discussion

Stem cell-based strategies are of particular interest in the study of neurological diseases because mature brains have a limited capacity for self-repair. MSCs have great potential to serve as therapeutic agents for stroke treatment, because they are easily obtained and can be expanded rapidly *ex vivo* for transplantation [9,10]. Transplanted MSCs in an ischemic region of the rat brain can differentiate into neural cells and promote functional improvement [11,12]. Furthermore, MSCs can improve neurological deficits in stroke patients [13]. hUCB-MSCs have proven to be more advantageous than bone marrow-derived MSCs in terms of cell procurement, storage, and transplantation [14]. Moreover, the number and differentiation ability of bone marrow-derived MSCs significantly decrease with age [15]. Thus, hUCB-MSCs are a better candidate for clinical applications of stem cell-based therapies.

In this study, we confirmed that intravenous infusion of hUCB-MSCs after transient MCAO in rabbits reduced the infarction volume, prevented ischemic neuronal damage, and improved neurological deficits. These results are consistent with previous studies in which beneficial effects were observed in cerebral ischemic models infused with MSCs [16-18].

^1^H-MRS enables serial, non-invasive monitoring of neuronal metabolite disturbances in the diseased brain *in vivo*, such as Cho, Cr, NAA, and Lac [6]. NAA is located primarily in neuroaxonal tissues. Its presence can be used to determine neuron numbers and activity. Therefore, decreased NAA indicates impaired neuronal and/or axonal function [8]. Cho is a marker of cell reversal and reflects the stability of cell membranes. Its peak level is usually related to cell membrane synthesis and catabolism, which depends on the concentrations of Cho in membrane phospholipids. Cho peaks can reflect the amount of Cho-containing compounds in the myelin membrane, such as phosphocholine and glycerophosphocholine. Abnormal Cho peaks can be observed in pathological conditions such as demyelination, degenerative changes, and ischemic states [19]. Lac is a redox partner of pyruvate and metabolic intermediate between glycolysis and Kreb’s or the tricarboxylic acid (TCA) cycle. When oxygen availability is low because of perfusion deficiency or other metabolic stress, the rate of the TCA cycle decreases and pyruvate produced by glycolysis accumulates and is converted to Lac. Thus, Lac can serve as a marker of anaerobic metabolism in stroke. Lac detection by ^1^H-MRS *in vivo* may allow identification of regions of metabolic stress in the brain and other human tissues, which can potentially identify regional ischemia in stroke [20]. Therefore, serial decreases in NAA/Cr and Cho/Cr ratios and elevations in the Lac/Cr ratio essentially indicate brain damage. In our current study, we observed an elevated Lac/Cr ratio and a marked decrease in NAA/Cr and Cho/Cr ratios at 24 h after MCAO in the MCAO group *(p* < 0.01), which was consistent with previous studies [18,21]. However, after intravenous delivery of hUCB-MSCs, ^1^H-MRS analysis at 24 h and 2 weeks indicated a significant decrease in the Lac/Cr ratio and a marked elevation in NAA/Cr and Cho/Cr ratios compared with those in MCAO and saline groups. These findings suggest neuroprotection induced by the transplantation procedure.

MSCs produce an impressive array of neuroprotective compounds. In a meta-analysis of 60 preclinical studies of intravenously delivered stem cells, Janowski and colleagues reported that good patient outcomes were correlated with inhibition of apoptosis [22]. Neuroprotection by the transplantation procedure may be direct via secretion of neuroprotective compounds or indirect via immunomodulation, angiogenesis, or amplification of the endogenous neural stem cell response [23]. In our present study, hUCB-MSC transplantation restored the ^1^H-MRS data to near normal at 2 weeks after MCAO. Moreover, hUCB-MSC transplantation protected neurons in the infarct area. Thus, our data may offer additional supportive evidence for neuroprotection by stem cells.

The safety of stem cell transplantation for treating ischemia is important. Short-term concerns include acute infusional toxicities to important organs, such as hepatotoxicity and renal toxicity. Long-term concerns include the risk of tumor formation [24]. In our present study, no influence was found on CBCs, liver function, renal function, serum glucose, or serum lipids in MCAO rabbits at 24 h and 2 weeks after transplantation.

## Conclusions

Taken together, our data show that transplanted hUCB-MSCs might ameliorate ischemic damage by influencing neuronal metabolites in the infarct area, which might offer additional supportive evidence for neuroprotection by stem cells. No significant changes were observed in CBCs or serum biochemical parameters, suggesting that intravenous infusion of hUCB-MSCs is safe for rabbits in the short-term.

## Methods

### Rabbit MCAO model

All animal protocols were approved by the Institutional Animal Care and Use Committee of Clinic College, Anhui Medical University. Male New Zealand white rabbits (provided by the Experimental Animal Center of Anhui Medical University, weighing 2.5–3.5 kg) were randomly divided into sham (n = 10), MCAO (n = 10), hUCB-MSC (n = 10), and saline (n = 10) groups. Anesthesia was induced with 2% sodium pentobarbital (20 mg/kg). The rectal temperature was maintained at 37°C throughout the surgical procedure as monitored by an electronic temperature controller linked to a heating pad (FHC, Bowdoinham, ME, USA). Transient MCAO was induced as described previously [25]. Briefly, the right common carotid artery (CCA), external carotid artery, and internal carotid artery (ICA) were exposed through a ventral midline incision. A nylon monofilament suture with a rounded tip (head-end diameter: 0.53 mm) was introduced into the CCA lumen and gently advanced into the ICA until it blocked the bifurcating origin of the MCA. At 2 h after occlusion, the rabbits were re-anesthetized and reperfused by withdrawing the suture until its tip cleared the lumen of the CCA.

### hUCB-MSC culture and identification

Ethical approval for the use of hUCB-MSCs was obtained from Clinic College, Anhui Medical University. Human UCB samples (60–80 ml) were collected from the umbilical vein of full-term infants during deliveries with informed maternal consent. Isolation and expansion of hUCB-MSCs was conducted as reported previously [26]. Briefly, mononuclear cells were isolated by density gradient centrifugation. The separated mononuclear cells were washed, resuspended in Dulbecco’s modified Eagle’s medium, and then seeded at 1 × 10^6^ cells/mL. Cultures were maintained at 37°C in a humidified atmosphere containing 5% CO_2_, and the culture medium was changed twice a week. Fibroblast-like adherent cells were observed in UCB-derived mononuclear cell cultures. After 1 –3 weeks, MSC colonies reached 80% confluence and the cells were trypsinized (0.25% trypsin), washed, resuspended in culture medium, and subcultured at 5 × 10^4^ cells/cm^2^. MSCs of each hUCB harvest were expanded *ex vivo* by successive subcultivation under the same conditions. Passage 5–8 cells with a more than 1,000-fold expanding capacity were used in experiments.

Isolated hUCB-MSCs were characterized by flow cytometric analysis of specific surface antigens using CD29-FITC, CD45-PE, CD44-FITC, and CD34-PE monoclonal antibodies (BD Bioscience, San Jose, CA, USA). The cells were incubated in the dark at room temperature for 20 min.

### Labeling of hUCB-MSCs with BrdU and tracing BrdU-positive cells in the injured brain

hUCB-MSCs at passage 5 were cultured in complete growth medium under the same conditions described above. Cells were labeled by incubation in medium containing 5 mmol/L BrdU (Roche Diagnostics, Mannheim, Germany) for 48 h before harvest. BrdU was added according to the manufacturer’s instructions. BrdU binds to DNA and labels the nucleus, serving as a tracer for *in vivo* studies of transplanted cells. The distribution of BrdU-positive cells was detected by immunohistochemical methods [27]. After 2 weeks, serial sections (6 μm thick) through the ischemic cortex were prepared from each rabbit. The sections were then incubated with a primary anti-BrdU monoclonal antibody (1:500) and then peroxidase-conjugated anti-rabbit IgG as the secondary antibody (1:100). The cells were stained with diaminobenzidine as the chromagen.

### hUCB-MSC transplantation

Passage 5 hUCB-MSCs were injected intravenously within several minutes after MCAO. hUCB-MSCs (5 × 10^6^) diluted in 2 mL PBS were injected slowly for 5 min via the right leg femoral vein. For the saline group, 2 mL PBS without hUCB-MSCs was injected in the same manner.

### Neurological deficit scale

Neurological deficits were measured in an independent and blinded fashion by NSSs as described by Purdy [28]. NSSs include motor function, consciousness, head turning, circling, and hemianopsia. The minimum NSS is 2, which is normal, and the maximal NSS is 11, indicating a comatose or dead animal. NSSs were measured at 6 h, 7 days, and 14 days after hUCB-MSC transplantation.

### ^1^H-MRS examination

^1^H-MRS data were obtained using a 1.5 Tesla MR machine (Siemens 1.5 T Novus MR scanner) and the point resolved echo spectroscopy method for localization and 5 mm × 5 mm × 5 mm volume of interest of the hippocampus in the bilateral temporal lobe. TE, TR, and NEX were 18, 400, and 200 ms, respectively. The ^1^H-MRS detection technology used was multi-voxel spectroscopy sequence of fixed-point resolution data acquisition. All spectral data were automatically processed in probe sequence on the MR console. Probe acquisition protocols employed in this study included ratio frequency transmitter receiver gain adjustment, water suppression, auto-shim process, and data acquisition. The area of the peak for each organic compound and its comparative value were obtained for each volume of interest. Four major peaks were measured individually and determined to be Cho (3.2 ppm), Cr (3.0 ppm), NAA (2.0 ppm), and Lac (1.3 ppm), which were measured individually. Peak areas were expressed as the relative ratio to Cr in each spectrum. Cr was used as the reference because of its relative stability, even under rapid energy metabolism fluctuations [29]. Infarct volumes were automatically calculated with Tools Free-hand ROI software (Siemens).Two neuroradiologists analyzed all ^1^H-MRS and MRI data.

### Histopathological studies

At 2 weeks after MCAO, all rabbits were anesthetized with 20% urethane and then their brains were quickly removed and fixed with 10% formalin. After 24 h, the tissues were embedded in paraffin and serial sections (6 μm thick) were prepared through the ischemic cortex. The sections were then stained with hematoxylin and eosin. Neurons in a linear length (1 mm) were counted under a light microscope.

### MAP2 immunohistochemistry

The sections were dewaxed with a standard procedure and washed with PBS as described previously [30]. Briefly, after blocking in 10% normal goat serum/PBS for 30 min at room temperature, the sections were incubated with a mouse anti-MAP-2 polyclonal antibody (1:200, Santa Cruz Biotechnology, Santa Cruz, CA, USA) for 16 h at 4°C. After three washes in PBS (10 min each), the sections were incubated with a goat anti-mouse secondary antibody for 2 h at room temperature. The sections were washed as described above and then incubated with an avidin biotinylated enzyme complex for 1 h at room temperature. The sections were visualized with diaminobenzidine. Nuclei were counterstained with hematoxylin. Finally, the sections were dehydrated in an alcohol gradient and cleared with xylene. Ten images were then randomly captured in microscopic fields for each group. The images were used to analyze the OD of MAP2-positive cells with JEDA 801D software (Jiangsu JEDA Science Technology Development, China).

### Statistical analysis

All data were expressed as the means ± standard error (SE). The Student’s t-test was used to compare differences between two groups. One-way analysis of variance was used to determine the significance of differences among multiple comparisons. Statistical significance was set at *p* < 0.05.

## Competing interests

The authors declare that they have no competing interests.

## Authors’ contributions

YM, YZ, ZW, and XC established the ischemia model, helped with sample preparation, measured NSSs, and performed histopathological studies and immunohistochemistry. BL performed cell cultures. YZ and HL measured the infarct area volume and performed ^1^H-MRS measurements. YM, YZ, HL, and QS drafted the manuscript. QS, and YZ conceptualized the study and participated in its design and coordination. All authors read and approved the final manuscript.
